# Obesity as a Catalyst for Endometrial Hyperplasia and Cancer Progression: A Narrative Review of Epidemiology, Molecular Pathways, and Prevention

**DOI:** 10.3390/biomedicines13112612

**Published:** 2025-10-25

**Authors:** Ionela-Mihaela Ordeanu, Cristina Jana Busuioc, Constantin-Cristian Văduva, Răzvan-Cosmin Pană, Ana-Maria Petrescu, Renata Maria Văruț, Mihaela Stanciu, Mihaela Popescu

**Affiliations:** 1 Doctoral School, University of Medicine and Pharmacy of Craiova, 200349 Craiova, Romania; mihaellaionella19@gmail.com; 2 Department of Histology, Faculty of Medicine, University of Medicine and Pharmacy of Craiova, 200349 Craiova, Romania; cristina.busuioc@umfcv.ro; 3 Research Center for Microscopic Morphology and Immunology, University of Medicine and Pharmacy of Craiova, 200349 Craiova, Romania; 4 Department of Gynecology, Faculty of Medicine, University of Medicine and Pharmacy of Craiova, 200349 Craiova, Romania; ana.petrescu048@gmail.com; 5 Faculty of Pharmacy, University of Medicine and Pharmacy in Craiova, 200349 Craiova, Romania; renata.varut@umfcv.ro; 6 Department of Endocrinology, Faculty of Medicine, Lucian Blaga University of Sibiu, 550169 Sibiu, Romania; mihaela.stanciu@yahoo.com; 7 Department of Endocrinology, Faculty of Medicine, University of Medicine and Pharmacy of Craiova, 200349 Craiova, Romania; mihaela.popescu.e@umfcv.ro

**Keywords:** endometrial hyperplasia, obesity, molecular pathways, genetic alterations, endometrial carcinoma

## Abstract

Obesity is a major, modifiable driver of endometrial carcinogenesis. This review distills how excess adiposity promotes malignant change and synthesizes prevention strategies across the hyperplasia–cancer continuum. Three converging axes underpin risk: aromatase-mediated estrogen excess; insulin resistance with hyperinsulinemia activating PI3K–AKT–mTOR signaling; and adipokine-driven low-grade inflammation with downstream NF-κB/STAT3 activity. Within this framework, EIN is the key precursor in which these pathways coalesce. Risk can be attenuated through progestin-based therapy (levonorgestrel-releasing intrauterine system or continuous oral regimens), structured weight management, and metabolic adjuncts in selected phenotypes (e.g., metformin for insulin resistance; incretin-based anti-obesity agents as emerging options). Bariatric surgery produces substantial weight loss and favorable metabolic shifts, though evidence for cancer risk reduction is largely observational. Overall, a practical precision-prevention approach—combining progestins with durable weight control and metabolic optimization under guideline-concordant surveillance—appears feasible in routine gynecologic care. Future research should establish causal effects, durability, and optimal sequencing/combination of interventions in trials with endometrial endpoints.

## 1. Introduction

With 420,242 new cases identified in 2022, endometrial cancer has now surpassed other gynecologic malignancies in prevalence across developed countries, and estimates predict an additional 50% increase in incidence by 2030 if current trajectories are maintained [1,2,3]. This alarming trajectory corresponds with the global spread of the obesity pandemic, highlighting excess adiposity as a key factor in endometrial carcinogenesis.

Expanded adipose tissue functions as an endocrine organ, promoting aromatase-mediated estrogen biosynthesis while also generating chronic systemic inflammation through dysregulated adipokine secretion. This hyperestrogenic state, combined with obesity-associated insulin resistance and hyperinsulinemia, activates key oncogenic signaling cascades such as phosphatidylinositol-3-kinase/protein kinase B/mammalian target of rapamycin (PI3K/AKT/mTOR) and Signal Transducer and Activator of Transcription 3 (STAT3), ultimately driving endometrial hyperplasia progression toward malignant transformation [4,5,6].

Based on these mechanistic insights, endometrial hyperplasia (EH) represents a critical transitional stage where preventive interventions may prove most effective. Atypical EH carries a lifetime progression risk of 20–50%, with annual rates rising from 8.2% at 4 years to 28% at 20 years. Early molecular events—such as PTEN loss (in up to 80% of cases), KRAS (Kirsten rat sarcoma virus) mutations in 10–30%, and microsatellite instability—map the stepwise evolution toward invasive carcinoma [7,8].

Despite obesity’s status as the principal modifiable risk factor, the integrated pathophysiological mechanisms linking adiposity to endometrial malignancy remain incompletely understood. Moreover, the optimal integration of lifestyle interventions, pharmacological therapies, and surgical approaches for women with EH and obesity requires systematic evaluation to develop evidence-based prevention strategies aimed at reducing the progression from endometrial hyperplasia to endometrial cancer.

**Figure 1:**
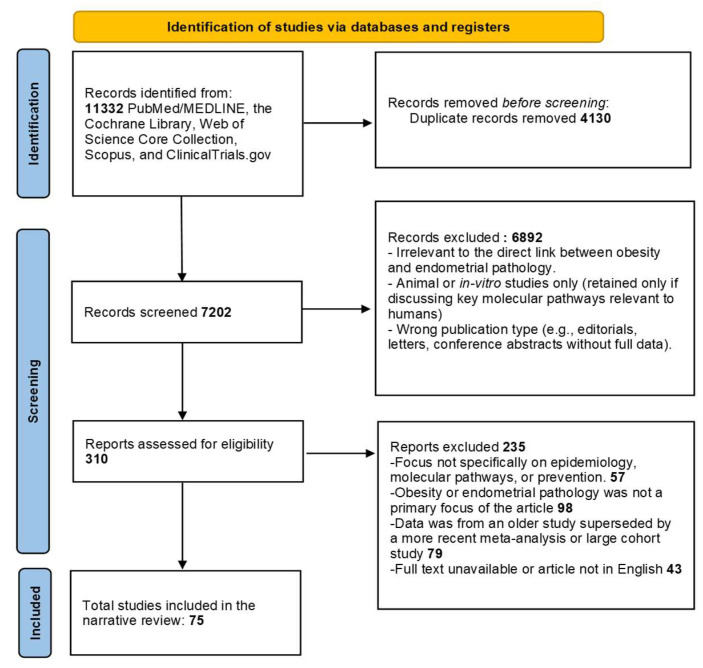
PRISMA 2020 Flow Diagram of the Study Selection Process. The diagram illustrates the systematic process of literature identification, screening, and final inclusion for this review. It details the number of records identified from databases and other sources, the removal of duplicates, the exclusion of articles during title/abstract and full-text screening, and the final count of studies included in the narrative synthesis.

Prior reviews established the epidemiologic and pathophysiologic links between obesity and endometrial cancer (EC) [1,2,3], summarized lifestyle and dietary determinants [1], and framed obesity-related implications across gynecologic malignancies, including preventive options such as bariatric surgery and the levonorgestrel-releasing intrauterine system (LNG-IUS) [2]. However, these reviews did not integrate endocrine–metabolic and inflammatory axes with tissue biomarkers—estrogen receptor (ER)/progesterone receptor (PR) status, progesterone resistance, insulin-like growth factor-1 (IGF-1) and its receptor (IGF1R)—into a single mechanistic-to-intervention framework, nor did they operationalize precision prevention through phenotype-guided pathways for women with obesity. In particular, they did not synthesize how estrogenic drive, insulin/IGF-1 signaling, and adipokine-mediated inflammation converge on PI3K/AKT/mTOR and Wnt/β-catenin pathways and epigenetic alterations that underlie endometrial intraepithelial neoplasia (EIN) and progesterone resistance, nor did they map where LNG-IUS/progestins, metabolic therapies, or bariatric surgery should be deployed along the endometrial hyperplasia (EH)→EIN→EC continuum. Our review addresses these gaps by (i) mapping obesity-related pathways onto endometrial remodeling and clonal evolution (EIN), (ii) aligning actionable intervention points (lifestyle, metabolic therapies, LNG-IUS/progestins, bariatric surgery) with the underlying biology, and (iii) proposing a biomarker-anchored precision-prevention algorithm specific to EC, including women with polycystic ovary syndrome (PCOS). This is timely because clinical practice is shifting toward risk-adapted prevention in high-risk obese populations, yet a consolidated, biomarker-anchored algorithm and figure that translate biology into care pathways are still lacking (see Figure 1).

Therefore, this review synthesizes epidemiological trends, molecular underpinnings, and evidence-based prevention strategies across the endometrial hyperplasia–cancer continuum. The review’s novelty lies in integrating updated epidemiological projections, offering an in-depth synthesis of molecular crosstalk (ER–mTOR and adipokine–IGF–insulin pathways), and framing prevention strategies within a precision, risk-stratified context.

## 2. Methods

Detailed searches were performed across PubMed/MEDLINE, the Cochrane Library, Web of Science Core Collection, Scopus, and ClinicalTrials.gov (restricted to interventional prevention studies). These databases were chosen to provide comprehensive coverage of both clinical and translational evidence (Figure 1).

The search strategy was structured around four core concept blocks, applied with Boolean operators and, where applicable, MeSH terms. The Population/Outcome block targeted endometrial pathology, using terms such as “Endometrial Neoplasms” [MeSH], endometrial cancer, endometrial carcinoma, and endometrial hyperplasia. The Exposure block captured obesity-related determinants, including obesity, “Body Mass Index”, BMI, and metabolic syndrome. For the Molecular focus set, additional terms reflecting mechanistic pathways were incorporated, such as pathway, mechanism, insulin, IGF, estrogen, aromatase, adipokine, inflammation, PI3K, AKT, and mTOR. For the Prevention set, intervention-related terms were included, such as bariatric, weight loss, lifestyle, metformin, GLP-1, semaglutide, and progestin.

Observational studies such as cohort and case–control designs were included, as well as meta-analyses and systematic or narrative reviews that provided relevant data. Basic and translational research papers elucidating obesity-linked pathways in the endometrium were also considered eligible. Furthermore, prevention and management studies were included which address lifestyle interventions, bariatric or metabolic surgery, and pharmacological approaches such as metformin, GLP-1 receptor agonists, or progestin-containing strategies, provided they reported outcomes related to endometrial hyperplasia (EH), endometrial cancer (EC), or validated risk biomarkers.

Exclusion criteria comprised non-human or pediatric studies, research on non-uterine cancers, and editorials or commentaries without primary data. Furthermore, case reports, studies without accessible full texts, duplicate publications were excluded as well as articles that did not specifically link obesity—or its treatment interventions—to EH/EC risk, underlying biology, or prevention.

Deduplication of records was performed using automatic filters based on title, author, and DOI, followed by manual verification. Screening was conducted in two stages: an initial review of titles and abstracts, followed by full-text assessment of potentially relevant articles. Reasons for excluding articles at the full-text stage included lack of relevant outcomes (no EH/EC endpoints), irrelevant exposures (not focused on obesity or obesity-targeted interventions), non-extractable or duplicated datasets, and publications not available in English.

## 3. Findings

### 3.1. Epidemiology

In developed nations, endometrial cancer (EC) is now the leading gynecologic malignancy, with 420,242 new cases in 2022 (2.1% of all cancers) and 97,704 deaths (1% of cancer mortality) [9]. In the United Kingdom, EC accounts for ~5% of new cancer cases and 8% of cancer deaths in women, with >9700 diagnoses annually, while the United States reports >65,000 new cases each year [1,2]. The Cancer Statistics 2024 report projects ~67,880 U.S. diagnoses in 2024 [10].

Age-standardized incidence shows marked geographical disparities that mirror economic development and adiposity patterns: high-income regions report about 28–29/100,000 versus ~3–4/100,000 in lower-middle-income settings [1]. Countries with the highest obesity prevalence closely parallel those with the highest EC incidence, supporting a strong epidemiologic link between excess adiposity and disease burden [11,12,13].

The rise is not confined to older women. Several populations report increasing diagnoses among premenopausal women, with annual percentage changes documented in low-grade tumors among those aged 30–39, consistent with shifts in lifestyle and metabolic exposures [13,14,15].

Attributable fraction and residual drivers. While obesity prevalence tracks closely with EC incidence, the proportion of cases attributable to adiposity varies by population and method. Contemporary estimates suggest that approximately one-third to one-half of EC in high-income settings is attributable to excess adiposity, with substantial heterogeneity across cohorts and modeling approaches; these values should be regarded as indicative rather than definitive given assumptions about causality, latency, and unmeasured confounding. Importantly, reproductive factors (nulliparity, early menarche, later menopause), hormone-therapy patterns (particularly unopposed estrogen), metabolic comorbidities (insulin resistance/type 2 diabetes), and socioeconomic determinants (including access to preventive care) contribute meaningfully to the residual increase not explained by adiposity alone. The BMI dose–response remains robust (≈60% higher risk per 5 kg/m^2^) although absolute risks and gradients differ across settings [3,16,17,18,19,20,21].

By 2050, demographic transitions will expand the global cancer burden, with a substantial share of the EC increase concentrated in regions undergoing rapid changes in adiposity and metabolic health. Notably, an estimated one-third of EC cases are potentially preventable through lifestyle modification and risk-directed care pathways, emphasizing the importance of obesity prevention, early detection, and hormonal management in high-risk groups [17,21,22,23,24].

The alarming epidemiological trends described above necessitate a deeper understanding of the premalignant conditions that precede invasive carcinoma, with endometrial hyperplasia representing the critical transitional state where preventive interventions may prove most effective in disrupting the pathway to malignancy.

#### 3.1.1. Role of Obesity in Endometrial Hyperplasia Progression

Obesity plays a critical role in both the development and progression of endometrial hyperplasia through multiple interconnected mechanisms. Obese women face a 2–4-fold higher risk of developing EIN and endometrial cancer compared to those with normal body weight. This elevated risk is mediated through three primary pathways: hormonal dysregulation, insulin resistance with hyperinsulinemia, and pro-inflammatory adipokine secretion [7].

The hormonal pathway involves adipose tissue facilitating the aromatization of androgens into estrogen, leading to chronic unopposed estrogen stimulation of the endometrial lining. This hormonal imbalance drives abnormal endometrial proliferation, and subsequent malignancy. Insulin resistance associated with obesity results in elevated insulin and insulin-like growth factor 1 (IGF-1) levels, which activate PI3K-AKT and (Mitogen-Activated Protein Kinase) MAPK signaling pathways critical for cell proliferation and survival. Hyperinsulinemia further exacerbates endometrial hyperplasia by reducing sex hormone-binding globulin (SHBG), thereby increasing bioavailable estrogen [10,11].

In premenopausal women presenting with abnormal uterine bleeding, studies reveal that nearly 5% harbor either endometrial cancer or complex hyperplasia on biopsy. Importantly, BMI proved more predictive of histopathological abnormalities than age, emphasizing that overweight and obese women warrant prompt evaluation even before menopause. Therapeutic interventions targeting weight reduction have demonstrated significant benefits, as following bariatric surgery, not only do biomarkers of insulin resistance improve rapidly, but histological resolution of atypical endometrial hyperplasia has also been documented, reflecting reversal of the neoplastic precursor state [12,25,26,27].

While endometrial hyperplasia represents the morphological precursor to cancer, the molecular drivers underlying this progression center on hormonal dysregulation. A key factor is the enhanced estrogen production from expanded adipose tissue, which creates a hormonal milieu conducive to both hyperplastic transformation and subsequent malignant evolution.

#### 3.1.2. Estrogen Biosynthesis: Aromatase in Adipose Tissue

In this context, adipose tissue serves as a critical site for estrogen biosynthesis through the activity of aromatase (CYP19A1), a key enzyme responsible for converting androgens into estrogens.

#### 3.1.3. Aromatase Expression and Activity in Adipose Tissue

In adipose tissue, aromatase primarily converts androstenedione, delivered from adrenal dehydroepiandrosterone (DHEA) and its sulfate, into estrone (E1). This differs from ovarian estrogen synthesis, where testosterone serves as the primary substrate for estradiol (E2) production. The efficiency of androstenedione-to-estrone conversion rises markedly with increasing age and adipose tissue mass, being more pronounced in women with a gynoid than an android fat distribution [28,29].

Moreover, adipose tissue possesses the ability not only to convert circulating steroid precursors but also to initiate de novo steroidogenesis from cholesterol. Although classical steroidogenic organs such as gonads, adrenals, and placenta are traditionally considered the main sites of de novo hormone synthesis, adipose depots predominantly depend on precursor conversion. Nevertheless, accumulating evidence indicates that adipose tissue is also capable of initiating steroidogenesis de novo, thereby extending its role beyond a passive conversion site [28,30].

#### 3.1.4. Depot-Specific Differences and Hormonal Regulation

Evidence indicates depot-specific differences in the adipose estrogen milieu, with higher estrone concentrations reported in visceral adipose tissue (VAT) than in subcutaneous adipose tissue (SAT) across both pre- and postmenopausal groups. These observations derive primarily from small, heterogeneous studies (often cross-sectional or ex vivo, with variable assays and tissue handling), so estimates should be interpreted with caution. In postmenopausal women with obesity, several cohorts have reported higher estradiol levels within VAT, whereas in premenopausal women obesity has been associated with increased CYP19A1 expression and a relative rise in estradiol synthesis in SAT—findings that are hypothesis-generating rather than definitive [28,31].

The regulation of aromatase activity depends on local substrate availability and is influenced by various factors including nutritional and menopausal status. Women generally exhibit higher estradiol concentrations in subcutaneous adipose tissue compared to men, correlating with increased expression of estrogen-converting genes. This sexual dimorphism in adipose tissue estrogen synthesis contributes to the observed differences in fat distribution patterns and metabolic profiles between sexes [28,30].

#### 3.1.5. Implications for Endometrial Cancer Pathogenesis

This peripheral estrogen production becomes the predominant source of estrogens in postmenopausal women, replacing ovarian synthesis and maintaining estrogen exposure even after natural menopause [1].

Chronic estrogen exposure drives endometrial carcinogenesis through multiple mechanisms, including the activation of estrogen receptor-alpha (ERα), which promotes endometrial epithelial cell proliferation by upregulating cell cycle regulators such as cyclin D1 and c-MYC. Unopposed estrogen stimulation also inhibits apoptosis through B-cell lymphoma 2 protein (BCL-2) upregulation while suppressing pro-apoptotic factors like BCL-2-associated X protein (BAX). The continuous estrogen exposure from adipose aromatase activity, particularly in the absence of adequate progesterone opposition, creates a permissive environment for malignant transformation [1,29,30,31,32].

Beyond local tissue effects, the increased estrogen production from adipose aromatase significantly impacts the hypothalamic-pituitary-ovarian axis. Elevated peripheral estrogen levels can disrupt normal gonadotropin release patterns, manifesting as menstrual irregularities including oligomenorrhea and menorrhagia. The feedback effects of adipose-derived estrogens on the hypothalamus and pituitary suppress normal cyclical hormone patterns, often resulting in anovulatory cycles that further reduce progesterone production and eliminate the protective effects of this hormone against estrogen-driven proliferation [33,34,35].

The endocrine disruption extends beyond estrogen production alone, as the expanded adipose tissue in obesity also alters the secretion of adipokines such as leptin and adiponectin. These adipokine imbalances compound the effects of hyperestrogenemia by interfering with normal ovarian steroidogenesis and creating a disrupted hormonal environment that compromises endometrial health. The resulting imbalance between estrogen and progesterone adversely affects the endometrial setting, creating conditions that may favor both benign hyperplastic changes and malignant transformation [33,36].

The chronic hyperestrogenemia generated through adipose aromatase activity establishes not only direct proliferative stimuli but also creates a pro-inflammatory microenvironment that amplifies carcinogenic potential through distinct molecular pathways that intersect with and magnify the hormonal effects previously described.

### 3.2. Chronic Inflammation-Molecular Mechanisms

#### 3.2.1. PI3K/AKT/mTOR Pathway Dysregulation in Obesity-Associated Endometrial Carcinogenesis

The molecular pathogenesis of obesity-associated endometrial cancer involves complex dysregulation of key cellular signaling networks, with the phosphatidylinositol-3-kinase (PI3K)/AKT/mechanistic target of rapamycin (mTOR) pathway serving as a central mediator of malignant transformation. Loss-of-function mutations in the tumor suppressor PTEN, occurring in 80–95% of Type I endometrial carcinomas, remove its antagonistic effects on PI3K, leading to constitutive AKT activation, enhanced cell survival, and resistance to apoptosis. Concurrently, activating mutations in the PIK3CA catalytic subunit amplify growth-factor responses via AKT1-PDPK1 cascades, further driving uncontrolled proliferation, while alterations in the PIK3R1 regulatory subunit facilitate aberrant membrane recruitment of PI3K in response to insulin and other ligands [1,4,36,37,38,39].

The mTOR signaling cascade represents a critical convergence point for obesity-related metabolic and hormonal perturbations in endometrial tissue. mTOR complex 1 (mTORC1) hyperactivation, evidenced by elevated phosphorylation of ribosomal protein S6 (pS6), drives typical endometrial hyperplasia through prolonged unopposed estrogen exposure. Genetic models demonstrate that uterine-specific deletion of PTEN recapitulates hallmark histological features of endometrial hyperplasia, while treatment with the mTOR inhibitor rapamycin reverses both pS6 accumulation and glandular overgrowth. In obesity-associated endometrial pathology, adipose-derived vascular endothelial growth factor (VEGF) engages VEGFR2 on endometrial cells, triggering Tyr1175 phosphorylation and subsequent mTORC1 activation, establishing a mechanistic link whereby obesity-associated angiogenesis amplifies mTOR-dependent proliferative signaling [4,39,40,41].

#### 3.2.2. Inflammatory Cytokine Networks and STAT3 Signaling

The chronic inflammatory milieu characteristic of obesity creates a microenvironment that can promote endometrial carcinogenesis via dysregulated cytokine signaling. Visceral adiposity is associated with sustained elevations of pro-inflammatory cytokines—particularly interleukin-6 (IL-6) and tumor necrosis factor-α (TNF-α)—which activate NF-κB and STAT3 pathways linked to cell survival and proliferation. Clinical studies have reported higher circulating IL-6 in endometrial adenocarcinoma (e.g., median 79.0 pg/mL vs. 31.0 pg/mL in controls) and greater STAT3 expression in tumors compared with benign hyperplasia; however, these findings come predominantly from small, tissue-level or cross-sectional datasets with heterogeneous assays and limited longitudinal follow-up [3,4,37,42].

STAT3 serves as a critical mediator of inflammation-driven endometrial carcinogenesis, with constitutive activation promoting transcription of genes involved in proliferation, inhibition of apoptosis, and immune evasion. Under physiological conditions, STAT3 activation remains transient and tightly regulated; however, in malignancy, persistent STAT3 signaling drives expression of anti-apoptotic genes including Bcl-xL, surviving, and Mcl-1, while simultaneously fostering immune escape mechanisms. Stratification by tumor differentiation reveals a stepwise increase in STAT3 expression across well-, moderately-, and poorly differentiated tumors, with median values rising from 0.74 to 1.43, correlating directly with advanced stage, deeper myometrial invasion, and presence of lymph node metastases [5,6,42].

#### 3.2.3. Adipokine Dysregulation and Metabolic Cross-Talk

Obesity-induced alterations in adipokine production create a complex network of metabolic and inflammatory signals that converge on endometrial tissue to promote carcinogenesis. Elevated leptin levels stimulate estrogen receptor α (ERα) and insulin-like growth factor-1 receptor (IGF-1R) signaling, while concurrent reductions in the anti-inflammatory adipokine adiponectin lead to diminished sex hormone-binding globulin (SHBG) synthesis and increased bioavailable estrogen activity. Resistin, another pro-inflammatory adipokine elevated in obesity, further exacerbates insulin resistance and activates inflammatory cascades that promote endometrial cell proliferation. The therapeutic significance of these alterations is demonstrated in bariatric surgery studies, where substantial weight loss (mean 30.15 kg over six months) results in coordinated improvements: SHBG, adiponectin, and insulin-like growth factor binding proteins increase significantly, while C-peptide, insulin, C-reactive protein (CRP), leptin, IL-1 receptor antagonist (IL-1Rα), and IL-6 decrease markedly [3,37,43].

The molecular mechanisms underlying adipokine-mediated endometrial pathology involve complex interactions between metabolic and growth signaling pathways. Leptin activates both PI3K/AKT and MAPK signaling cascades, promoting cell survival and proliferation while simultaneously inducing angiogenesis through VEGF upregulation. Hypoxia-inducible factor 1α (HIF-1α) stabilization in the adipose tissue microenvironment further amplifies VEGF expression and glycolytic enzyme activity, supporting tumor growth and vascularization. Reactive oxygen species generated by inflammatory cells in the obesity-associated microenvironment inflict cumulative DNA damage, which, in the context of impaired DNA repair mechanisms, drives progressive carcinogenesis [1,3,36].

Thus, low-grade chronic inflammation emerges as the pivotal pathogenic link interconnecting obesity, insulin resistance, and hormonal disturbances, decisively contributing to endometrial remodeling and the progression toward hyperplasia and carcinogenesis.

#### 3.2.4. mTOR–Estrogen Cross-Talk, Translational Control, and Contextual Modifiers

The bidirectional communication between estrogen receptors and mTOR is a core mechanism in obesity-associated endometrial pathology. ERα/β engage both genomic and non-genomic dialogues with PI3K/AKT/mTOR: ligand-bound ERs transcriptionally upregulate growth-factor ligands, receptor tyrosine kinases, and adaptor proteins that activate PI3K, thereby sensitizing endometrial cells to mitogenic inputs. Non-genomically, ERα associates with the PI3K p85α regulatory subunit and with the mTORC1 scaffold Raptor, enhancing kinase signaling and nuclear substrate phosphorylation independent of transcription [4,36,44]. Reciprocally, the mTORC1 effector S6K1 phosphorylates ERα at Ser167, augmenting ER transcriptional potency and establishing a feed-forward loop. Estrogen also amplifies mTOR outputs via IGF-1R upregulation, sustaining proliferative/survival programs that are accentuated in obesity-related endocrine milieus (increased aromatization, reduced SHBG) [37,45]. This integrated ER↔mTOR circuit helps explain the heightened estrogenic vulnerability of the endometrium in obesity and provides mechanistic support for combinatorial targeting of ER and mTOR pathways in EIN and early EC [4,36,44].

Beyond linear activation, mTOR operates within multilayered regulatory networks shaped by metabolic and stress signals that are common in obesity and PCOS. Hyperandrogenism can suppress AMPK—an upstream brake on mTORC1 through TSC1/2 and direct Raptor inhibition—while insulin resistance/hyperinsulinemia drive PI3K/AKT-dependent mTORC1 activation, jointly unleashing translational programs and attenuating FOXO-mediated restraint [4,36,46]. Endoplasmic-reticulum stress further modulates this landscape: activation of the PERK–eIF2α axis and CHOP typically antagonizes mTORC1 to promote apoptosis, yet in hyperplastic endometrium chronic, unresolved ER stress may become maladaptive, fostering dysregulated protein synthesis, incomplete autophagy, and survival under proliferative pressure [4,6,36,46]. Collectively, these inputs position ER–mTOR cross-talk and translational control as convergence nodes through which hormonal, metabolic, and stress cues promote progesterone resistance and disease progression—thereby offering a coherent rationale for pairing local progestins with metabolic interventions (weight loss, insulin-sensitizers/GLP-1RA) in appropriately selected patients.

### 3.3. Insulin Resistance and Metabolic Dysregulation: Central Drivers of Endometrial Pathogenesis

#### 3.3.1. Hyperinsulinemia and Direct Mitogenic Effects on Endometrial Tissue

Insulin resistance emerges as a fundamental metabolic derangement that directly promotes endometrial carcinogenesis through distinct molecular mechanisms beyond inflammatory pathways. Hyperinsulinemia characteristic of obesity and metabolic syndrome exerts direct mitogenic effects on endometrial cells through activation of specific insulin receptors identified in both normal endometrial tissue and malignant cells, triggering robust phosphorylation of insulin receptor substrate (IRS) proteins and subsequent PI3K/AKT pathway activation. This insulin-mediated signaling cascade promotes enhanced cell survival, increased glucose uptake, and accelerated protein synthesis, creating a cellular environment conducive to uncontrolled proliferation.

The synergistic interaction between IGF-1 and estrogen signaling creates a particularly potent mitogenic environment that exemplifies the convergence of metabolic and hormonal dysfunction in endometrial carcinogenesis. IGF-1 pathway activation amplifies estrogen receptor transcriptional activity through PI3K-mediated phosphorylation cascades, while estrogen upregulates IGF-1 receptor expression, establishing a feed-forward amplification loop that sustains endometrial hyperplasia and facilitates progression toward malignant transformation. This molecular synergy becomes especially pronounced when occurring in the context of common genetic alterations characteristic of Type I endometrial carcinoma: PI3K mutations, particularly activating mutations in PIK3CA encoding the catalytic subunit, synergize with PTEN tumor suppressor loss to create a molecular environment where IGF-1 and insulin signaling become pathologically amplified. PTEN normally functions as a critical negative regulator of PI3K signaling; its inactivation in 80–95% of Type I endometrial carcinomas removes this crucial brake on pathway activity, leading to constitutive AKT activation that, when combined with hyperinsulinemia and IGF-1 pathway hyperactivity, drives aggressive cellular transformation characterized by uncontrolled proliferation, resistance to apoptosis, and enhanced survival signaling [1,4,36]. The pathological significance of this direct insulin action is amplified by its ability to activate insulin-like growth factor receptors through cross-reactivity, creating a dual-receptor engagement that magnifies mitogenic signaling beyond what either pathway could achieve independently [1,47,48,49].

The systemic metabolic consequences of hyperinsulinemia extend beyond direct cellular effects to encompass fundamental alterations in sex hormone homeostasis that create a pro-carcinogenic endocrine environment. Elevated insulin levels suppress hepatic synthesis of sex hormone-binding globulin (SHBG), dramatically increasing bioavailable estrogen concentrations and establishing a hormonal milieu that synergizes with direct insulin signaling to promote endometrial proliferation. This dual mechanism—direct mitogenic stimulation coupled with enhanced estrogen bioavailability—creates a particularly potent oncogenic environment where metabolic dysfunction and hormonal excess converge to foster endometrial hyperplasia and subsequent malignant transformation [29,33,47,48].

The suppression of sex hormone-binding globulin (SHBG) synthesis represents a critical mechanism whereby insulin resistance amplifies estrogenic stimulation of endometrial tissue, creating a hormonal environment that synergizes with direct metabolic effects to promote carcinogenesis. By reducing hepatic SHBG production, hyperinsulinemia sustains elevated levels of bioavailable estrogens that far exceed what would be expected from obesity-associated aromatase activity alone. This mechanism creates a molecular environment where relatively modest increases in total estrogen production are magnified into substantial elevations in biologically active hormone levels, particularly in postmenopausal women where endogenous estrogen production is already low. The pathological significance of SHBG suppression extends beyond simple hormone availability to encompass fundamental alterations in estrogen receptor signaling dynamics, where increased ligand availability enhances receptor occupancy and transcriptional activity in endometrial cells [4,33,50].

The clinical significance of SHBG suppression is illustrated by weight loss interventions that simultaneously target multiple components of the metabolic–hormonal axis. Improved insulin sensitivity, SHBG restoration, and reestablishment of hormonal balance collectively reduce endometrial cancer risk. This comprehensive metabolic recovery demonstrates how addressing insulin resistance yields therapeutic benefits that extend beyond glucose control, encompassing fundamental improvements in the hormonal microenvironment that drives endometrial pathogenesis [4,33,50].

#### 3.3.2. Metabolic Reprogramming and Glucose Handling Dysfunction

Insulin resistance-associated endometrial pathology involves fundamental alterations in cellular glucose metabolism that extend beyond simple pathway activation to encompass sophisticated reprogramming of energy utilization and biosynthetic capacity. The molecular basis of these metabolic alterations centers on dysregulated insulin receptor/PI3K/AKT/mTOR signaling cascades that normally coordinate glucose homeostasis with cellular growth demands. In insulin resistance states, this coordination becomes severely disrupted, leading to impaired glucose uptake evidenced by reduced GLUT-4 expression in endometrial tissue compared to normal endometrium. This metabolic dysfunction creates a cellular environment where energy substrate availability becomes mismatched with proliferative demands, fostering compensatory adaptations that promote survival and growth under metabolically stressed conditions [4,29,39,51,52].

The therapeutic restoration of metabolic balance through interventions targeting glucose handling demonstrates the tractability of these pathways for endometrial cancer prevention.

**Figure 2:**
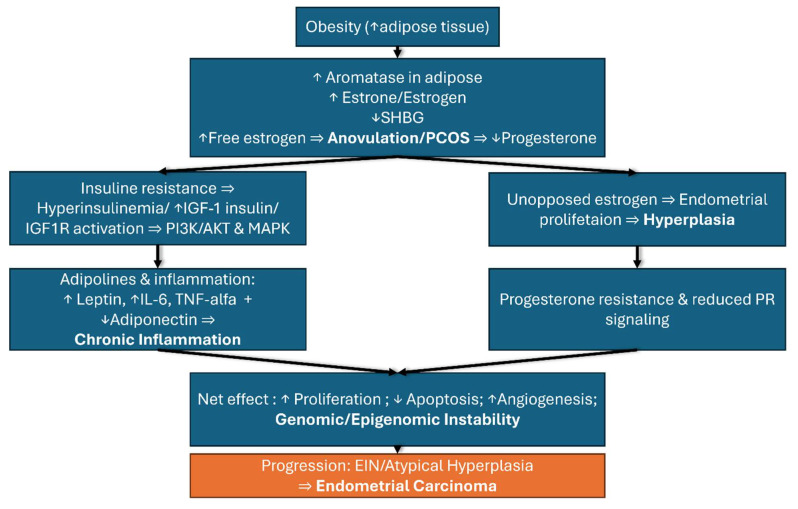
Obesity as a factor for endometrial cancer: metabolic and hormonal pathways.

**Figure 3:**
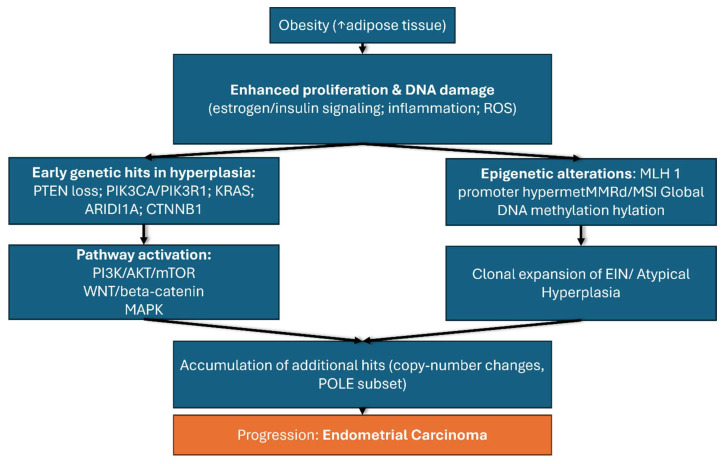
Obesity as a factor for endometrial cancer: genetic pathways.

Obesity represents one of the most important risk factors for the development of endometrial cancer through a complex interplay of hormonal, metabolic, and genetic mechanisms that occur simultaneously and reinforce each other. These intricate and vast reactions cannot be understood in isolation but rather as a network of interdependent processes that together explain the strong association between obesity and endometrial cancer. For clarity, these mechanisms are summarized in Figure 2, which illustrates the metabolic and hormonal pathways, and Figure 3, which presents the key genetic alterations involved.

### 3.4. Prevention Strategies

**Figure 4:**
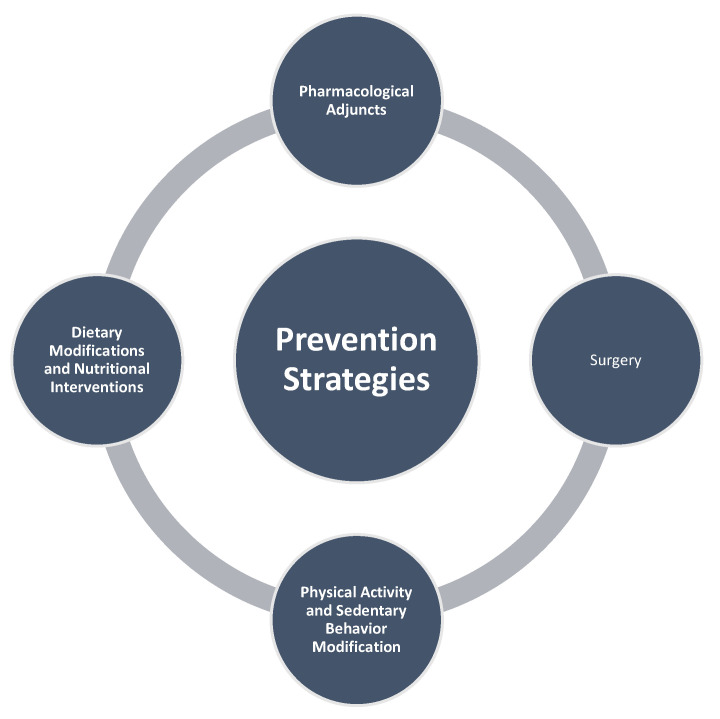
Main targets of prevention strategies.

**Figure 5:**
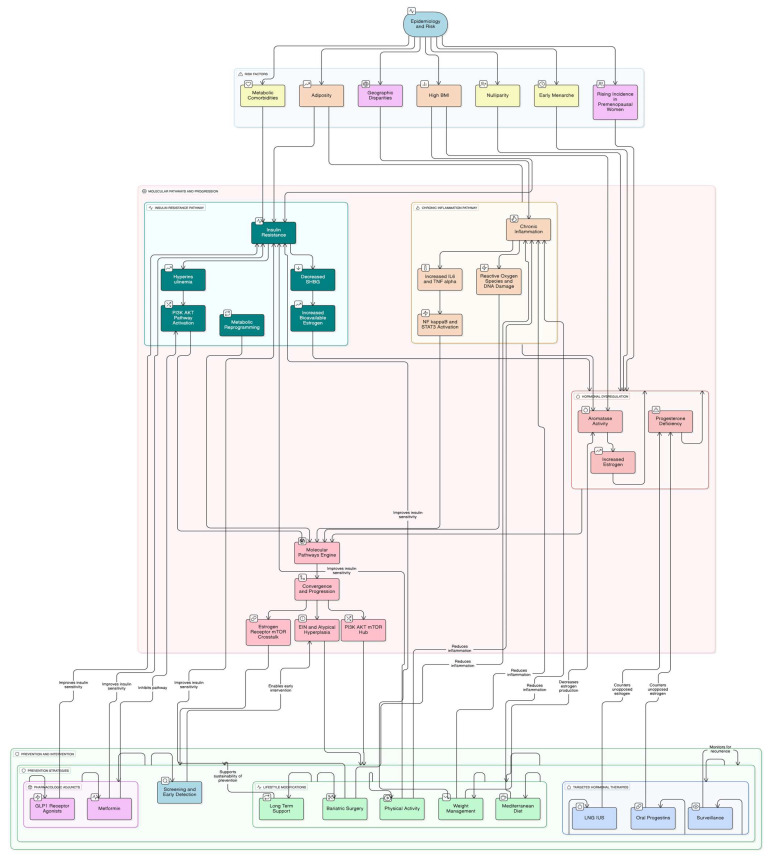
Obesity and the Endometrium: Mechanisms and Precision-Prevention Targets. The map links adiposity-driven endocrine (aromatase-mediated estrogen excess/unopposed estrogen), insulin–IGF-1, and inflammatory/adipokine axes to convergence on progesterone resistance, dysregulated proliferation/apoptosis, and genomic/epigenomic instability, leading to early events (PTEN, PIK3CA/PIK3R1, KRAS, ARID1A, CTNNB1; MLH1 hypermethylation/MMRd), EIN, and progression to EC. Intervention nodes are overlaid where they act: lifestyle/weight loss (including bariatric surgery), metabolic adjuncts (metformin; GLP-1/GIP–GLP-1 receptor agonists), and endometrial hormonal therapy (LNG-IUS or continuous oral progestins), with close histologic surveillance at EIN.

The substantial contribution of obesity to endometrial cancer risk presents significant opportunities for primary prevention through targeted lifestyle interventions. Given that over 60% of endometrial cancers are preventable through weight management, physical activity, and hormonal therapies, comprehensive lifestyle modifications represent a cornerstone of population-level cancer prevention strategies (Figure 4 and Figure 5) [21,23].

Even modest weight loss provides measurable protection against endometrial cancer. Evidence indicates that intentional weight reduction is associated with a lower risk, with relative risk values ranging between 0.61 and 0.96, corresponding to decreases in risk of up to 39%. Greater benefits appear to be achieved with higher levels of weight loss [53]. Clinical evidence demonstrates that patients achieving more than 3% weight loss show significantly higher reversal rates of endometrial hyperplasia compared to those with minimal weight loss (91.2% vs. 77.6%, *p* = 0.034), establishing weight reduction as an independent protective factor [21,22]. Meta-analysis of weight loss interventions reveals that across 29 studies, the pooled average weight loss was 13.8%, with bariatric surgery achieving the greatest reduction (25.8%). These interventions demonstrate significant effects on obesity-related biomarkers linked to endometrial cancer risk, including substantial reductions in inflammatory markers and hormonal mediators [54].

While bariatric surgery provides the most dramatic weight reductions, lifestyle interventions and pharmacotherapy offer accessible alternatives for broader populations. Lifestyle modifications, though achieving more modest weight loss (average 5.9%), remain foundational and broadly implementable approaches. Pharmacotherapies such as semaglutide offer intermediate benefits with moderate weight reduction (average 7.6%), while providing substantial improvements in key biomarkers including up to 40% decreases in leptin levels and meaningful reductions in inflammatory markers [54,55].

#### 3.4.1. Dietary Modifications and Nutritional Interventions

Dietary patterns significantly modulate endometrial cancer risk through effects on body composition, metabolic milieu, and hormone metabolism. Diets high in total lipids, saturated fats, and cholesterol correlate with increased risk by augmenting peripheral estrogen synthesis and systemic inflammation, while red meat consumption has been associated with approximately 60% higher risk, potentially due to carcinogenic compounds generated during high-temperature cooking and heme iron content [1].

Conversely, adherence to a Mediterranean dietary pattern, rich in olive oil, fish, and plant-based foods, demonstrates protective effects with up to 50% risk reduction. High-glycemic foods and elevated sugar intake may modestly raise endometrial cancer risk via hyperinsulinemia and insulin-like growth factor signaling, particularly in women not using hormone replacement therapy [1,56,57].

Specific nutritional components demonstrate promising chemopreventive properties. Coffee consumption, particularly filtered varieties rich in chlorogenic acid, has been linked to 20–35% decreases in endometrial cancer risk through antioxidant, anti-inflammatory, and insulin-sensitizing actions [58]. Soy isoflavones and lignans exhibit weak estrogenic and anti-estrogenic effects, with case–control studies suggesting approximately 20% risk reduction. Micronutrients with antioxidant properties, including carotenoids, vitamins C and E, and selenium, demonstrate modest inverse associations, while calcium intake may confer protective effects by binding bile acids and oxidants in the gut [1,56,59,60].

Adherence to dietary interventions improves when counseling is brief, structured, and repeatable, making it feasible within routine gynecologic visits. A short 5–10 min discussion can outline realistic goals (typically 5–10% of baseline weight linked to reduced endometrial risk), explore barriers, and agree on one achievable step such as adopting a Mediterranean-style plate, replacing sugary drinks, or increasing daily fiber intake. Same-day referral to dietetics or weight-management services enhances implementation, while follow-up can be aligned with scheduled gynecologic evaluations (e.g., endometrial sampling every 3–6 months). Low-burden supports—including self-monitoring, weekly self-weighing, or brief nurse contact—have been shown to improve retention without increasing clinic time. Where appropriate, adjunct pharmacotherapy may complement lifestyle measures. Recording weight, waist circumference, and a simple adherence marker in the gynecologic record helps sustain continuity across visits and supports team-based care.

#### 3.4.2. Physical Activity and Sedentary Behavior Modification

Regular physical activity provides substantial protection against endometrial cancer through multiple mechanisms. Engaging in at least 150 min per week of moderate exercise lowers risk by 30–40%, likely through weight control, increased sex hormone-binding globulin levels, and reduced bioavailable estrogen. Physical activity and diets rich in antioxidants may lower circulating insulin-like growth factor 1 and insulin, hormones implicated in carcinogenesis [1,61,62].

Conversely, sedentary behavior significantly increases endometrial cancer risk across multiple domains. High levels of overall sedentary time are associated with a 28% increased risk, with domain-specific analyses revealing 22% higher risk for occupational sitting and 55% higher risk for total daily sitting. Sedentary behavior exacerbates risk by reducing sex hormone-binding globulin levels, elevating free estradiol, and promoting insulin resistance and systemic inflammation—all of which synergize with estrogenic signaling to drive endometrial neoplasia [1,63,64].

Integrated approaches that combine multiple lifestyle factors demonstrate superior protective effects compared to single interventions. Greater adherence to comprehensive cancer prevention guidelines is associated with substantial risk reduction, with pooled analysis showing a hazard ratio of 0.54 (95% CI: 0.40–0.73) for highest versus lowest adherence categories. Dose–response meta-analysis demonstrates a 6% reduction in endometrial cancer risk per one-point increase in adherence score, highlighting the cumulative benefits of multiple lifestyle modifications [61,62].

Long-term maintenance of lifestyle-induced weight loss remains challenging in routine care; weight regain within 1–3 years is common and may begin by ~36 weeks after the end of active treatment [65]. Maintenance improves with continued, low-burden support—periodic follow-up, self-monitoring, and eHealth adjuncts—although effect sizes are modest and heterogeneous across reviews [66]. By contrast, in highly structured programs such as Look AHEAD, sustained differences in weight and body composition were still detectable 12–16 years after randomization—mainly in men—highlighting a gap between intensive trial conditions and routine practice [67]. Accordingly, estimates of diet-associated risk reduction should be interpreted in light of maintenance challenges, and prevention programs should include explicit relapse-prevention and re-engagement pathways. Embedding brief, structured dietary support within routine gynecologic follow-up may improve durability and translate metabolic gains into sustained reductions in endometrial risk.

#### 3.4.3. Pharmacological Adjuncts to Lifestyle Interventions

Evidence for metformin in EIN now includes both non-randomized cohorts and a small randomized trial. In a prospective single-center cohort of premenopausal patients treated with progestin (metformin prescribed only in insulin resistance), histologic reversal was 93.2% (55/59) with metformin versus 52.4% (11/21) without (*p* < 0.001), with multivariable analysis indicating an independent association with reversal; >3% weight loss was also independently associated (follow-up every 3–6 months; median ≈ 17.5 months). In parallel, a double-blind, placebo-controlled RCT in non-atypical EH (megestrol acetate 40 mg/day for 14 days/month) reported 93.1% histologic remission at 3 months with adjunct metformin 1000 mg/day (29/29 analyzed) versus 70.4% with placebo (19/27 analyzed). Taken together, these findings support metformin as a promising adjunct to progestin, particularly in insulin-resistant phenotypes; however, effect sizes should be interpreted cautiously given the small sample sizes, short follow-up in the RCT, single-center design and indication bias in the cohort, and limited data on recurrence and EIN. Mechanistically, preclinical work suggests AMPK activation, mTOR inhibition, and progesterone-receptor upregulation, but clinical causal mediation remains to be determined [22,55,68].

The evidence collectively supports a multi-modal approach to endometrial cancer prevention, with lifestyle interventions forming the foundation of risk reduction strategies. While sustaining substantial long-term weight loss through diet and exercise alone presents challenges for many high-risk individuals, the demonstrated benefits of even modest weight reduction (>3%) provide achievable targets for clinical intervention. Technology-based weight loss interventions, including text messaging applications, have achieved average losses of 4.4 kg and enhanced well-being in high-risk populations, suggesting scalable approaches for broader implementation [1].

Incretin-based anti-obesity therapies (e.g., GLP-1 receptor agonists such as semaglutide; dual GIP/GLP-1 agonists such as tirzepatide) improve adiposity, insulin resistance, and inflammatory profiles, providing a biologically plausible pathway for endometrial risk modification [69]. Large real-world analyses suggest an association between GLP-1RA exposure and lower incidence of several obesity-associated cancers, including endometrial cancer (GLP-1RA vs. insulin, HR 0.74; 95% CI 0.60–0.91), while showing no overall advantage vs. metformin—signals that remain observational and potentially affected by allocation/immortal-time bias and limited site-specific power [69,70]. A recent meta-analysis of randomized tirzepatide trials (26–72 weeks) indicates no increase in overall or site-specific cancer risk, supporting short- to mid-term oncologic safety but not establishing chemopreventive efficacy [71]. Preclinical endometrial models show synergy between semaglutide and progestins (e.g., levonorgestrel), with upregulation of PR and PGRMC1/2 and greater cytotoxicity than either agent alone—hypothesis-generating data that merit clinical testing [72]. Methodologically, current observational signals require cautious interpretation given recurrent pitfalls (confounding by adiposity/indication, immortal-time and survival biases, inadequate latency, and dose–response issues); target-trial emulations and prospective trials with endometrial endpoints (EH/EIN/EC) are needed [70]. Overall, incretin therapies should be framed as adjunct metabolic risk-modifiers—particularly in insulin-resistant phenotypes—that may complement weight loss and progestin-based strategies, rather than stand-alone chemopreventives, pending higher-quality causal evidence.

Bariatric surgery has emerged as a highly effective intervention for reducing endometrial cancer risk in women with severe obesity, with accumulating evidence demonstrating substantial protective effects that extend well beyond conventional weight management approaches. The Swedish Obese Subjects (SOS) Study, one of the most extensive and long-term prospective trials in this area, monitored 1420 women who had undergone bariatric surgery alongside 1447 controls managed conventionally for obesity, over a median follow-up of 18.1 years. Findings from this pivotal trial indicated that bariatric surgery conferred a 32% decrease in the overall risk of female-specific cancers (HR = 0.68; 95% CI 0.52–0.88; *p* = 0.004), with an even stronger 44% reduction observed in endometrial cancer incidence (HR = 0.56; 95% CI 0.35–0.89; *p* = 0.014). The analysis further estimated that 56 women would need to undergo bariatric surgery to prevent one case of female-specific cancer within a decade [5].

The protective effects of bariatric surgery appear to be most pronounced in women with baseline metabolic dysfunction, particularly those with hyperinsulinemia. Interaction analyses from the Swedish cohort demonstrated that women in the medium and high baseline insulin tertiles experienced greater relative risk reduction (HRs 0.62 and 0.57, respectively) compared with those in the low insulin tertile (*p* for interaction = 0.022), indicating that metabolic dysregulation amplifies the protective effect of surgical weight loss against female-specific cancers. This finding suggests that insulin levels, rather than adiposity alone, may be the critical determinant of cancer risk stratification, highlighting the importance of metabolic phenotyping in identifying optimal candidates for bariatric intervention.

Beyond effectiveness, eligibility, access, and long-term follow-up determine the real-world impact of bariatric surgery. Current criteria recommend surgery for BMI ≥ 35 kg/m^2^ regardless of comorbidity and allow consideration at BMI 30–34.9 kg/m^2^ in the presence of metabolic disease or when durable non-surgical weight loss has failed (per ASMBS/IFSO 2022; align with local policies). Early referral to an accredited multidisciplinary service helps navigate coverage restrictions, wait-lists, geographic constraints, and pre-operative requirements (nutrition and psychological assessment). Lifelong follow-up is essential: micronutrient supplementation and surveillance (iron, B12, folate, vitamin D, calcium), weight-trajectory and metabolic monitoring, contraception counseling and avoidance of pregnancy for 12–18 months, bone-health vigilance, and strategies to prevent weight regain. Post-operative visits at 3, 6, and 12 months, then annually can be synchronized with gynecologic surveillance when indicated. Because most risk-reduction data derive from observational cohorts with potential residual confounding, surgery should be framed as part of a personalized, risk-stratified pathway, not a universal solution [73,74,75].

The observed risk reductions (~32–62%) derive largely from prospective but non-randomized cohorts (e.g., SOS) and remain susceptible to selection/indication bias and residual confounding despite multivariable adjustment [5]. Time-varying factors (weight cycling, changes in diabetes therapy), informative censoring, and procedure heterogeneity (Roux-en-Y vs. sleeve gastrectomy; secular changes in care) may further influence estimates. Importantly, differential hysterectomy rates after bariatric surgery reduce the population at risk for endometrial cancer and can artifactually lower incidence if not fully accounted for. Consequently, reported HRs and NNTs should be interpreted as upper-bound associations rather than causal magnitudes; the stronger signals in hyperinsulinaemic phenotypes are biologically plausible but require confirmation with target-trial emulations or randomized settings.

#### 3.4.4. Magnitude of Risk Reduction and Meta-Analytic Evidence

The protective effect of bariatric surgery against endometrial cancer has been consistently demonstrated across multiple populations and study designs. A comprehensive meta-analysis of eight cohort studies encompassing 346,430 surgical patients versus 1,075,024 controls revealed that bariatric surgery confers a remarkable 62% reduction in endometrial cancer risk (RR 0.38, 95% CI 0.26–0.55, *p* < 0.00001). Notably, the divergence in cancer-free survival curves emerged after six years of follow-up, underscoring the requirement for sustained metabolic improvements to effect endometrial risk reduction. Sensitivity analyses excluding individual studies sequentially yielded consistent risk estimates (RR range 0.36–0.42), confirming the robustness of these findings [76].

#### 3.4.5. Mechanistic Foundations of Cancer Risk Reduction

The cancer-protective effects of bariatric surgery stem from comprehensive improvements in multiple interconnected pathways implicated in endometrial carcinogenesis. Surgical weight loss leads to rapid and sustained decreases in circulating leptin levels, with documented reductions from 60.2 ± 14.3 ng/mL to 12.5 ± 9.4 ng/mL at 12 months, accompanied by a doubling of adiponectin concentrations. This fundamental reshaping of the adipokine milieu creates an anti-inflammatory, anti-proliferative environment that disrupts key tumor-promoting signaling cascades. Concurrently, bariatric surgery substantially reduces both visceral and subcutaneous adipose tissue, which in turn leads to lower aromatase-driven estrogen production. This results in significant decreases in circulating estradiol levels, particularly in older women (median drop from 53.9 pg/mL to 35.7 pg/mL at 12 months), thereby mitigating the unopposed estrogenic stimulation that drives endometrial proliferation [76].

#### 3.4.6. Biomarker Normalization and Inflammatory Modulation

Longitudinal investigations of biomarker changes following bariatric surgery demonstrate comprehensive normalization of endometrial cancer risk factors within six months post-operatively. Multiple pro-inflammatory cytokines and insulin resistance markers decrease substantially, while protective binding proteins increase, with several post-operative levels approaching those observed in non-obese women. These changes include significant reductions in C-reactive protein (average decrease 47% with bariatric surgery), interleukin-6 (46.3% reduction), and improvements in insulin sensitivity reflected by lower HOMA-IR scores and glycosylated hemoglobin levels. The magnitude of biomarker improvement correlates with the extent of weight loss, with bariatric surgery achieving superior inflammatory marker reductions compared to lifestyle interventions or pharmacotherapy [43,54].

#### 3.4.7. Targeted Hormonal Therapies (Progestins)

Targeted hormonal therapies, particularly progestins, represent a cornerstone of endometrial cancer prevention strategies, especially in high-risk populations including obese women. The rationale for progestin use stems from the fundamental role of unopposed estrogen in endometrial carcinogenesis, where estrogen stimulation without adequate progesterone opposition leads to endometrial hyperplasia and subsequent malignant transformation. Over 60% of endometrial cancers are preventable through strategic interventions including weight management, physical activity, and targeted hormonal therapies, with progestins playing a central role in this preventive approach [77].

The levonorgestrel-releasing intrauterine system (LNG-IUS) has emerged as one of the most effective targeted prevention strategies, offering direct endometrial progestin delivery that reduces cancer risk by approximately 80%. This remarkable efficacy is achieved through direct suppression of endometrial proliferation, reversal of hyperplastic changes, and suppression of Ki-67 proliferation markers. Unlike systemic hormonal therapies, the LNG-IUS provides targeted local delivery while avoiding systemic side effects, making it particularly suitable for obese women who may have contraindications to oral hormonal therapies due to increased thrombotic risks [77,78].

Multiple progestin formulations have demonstrated robust endometrial protection across various regimens and delivery routes. Natural micronized progesterone and synthetic agents including dydrogesterone, medroxyprogesterone acetate (MPA), norethisterone acetate, and drospirenone show excellent protection in both continuous and sequential regimens. Notably, the FDA endometrial safety criteria have been fulfilled for numerous formulations, including sequential and continuous oral micronized progesterone, dydrogesterone, MPA, and various delivery routes of norethisterone acetate and levonorgestrel. These agents demonstrate hyperplasia incidence rates consistently below 1%, with some studies reporting complete absence of endometrial malignancy during treatment periods [79,80,81].

Network meta-analysis of 21 randomized controlled trials involving 2276 women revealed that high-potency progestin combined with metformin achieved the best outcomes for endometrial complete regression, while MPA emerged as the most effective single-agent therapy. The LNG-IUD demonstrated significantly higher complete regression rates compared to norethisterone, reinforcing its position as a first-line intervention for hyperplasia treatment and, by extension, cancer prevention [11,79,82].

Combination approaches integrating progestins with metabolic interventions show enhanced efficacy compared to hormonal therapy alone. Even modest weight loss exceeding 3% significantly improves treatment outcomes, with combined progestin and weight loss strategies showing superior results compared to progestin monotherapy [22].

Long-term use of combined oral contraceptives represents another effective hormonal prevention strategy, with prolonged use (≥20 years) associated with a 67% reduction in endometrial cancer risk. This protective effect persists for decades after discontinuation and remains independent of BMI, making it particularly relevant for obese women. However, obesity often limits eligibility for oral contraceptive use due to increased thrombotic risks, highlighting the importance of alternative delivery methods such as the LNG-IUS for high-risk populations [78,81].

International guidance converges on a conservative, selection-dependent approach. Suitable candidates are patients who wish to preserve fertility and have EIN or grade-1 endometrioid carcinoma without myometrial invasion, after rigorous exclusion of concurrent carcinoma by expert pathology with targeted, preferably hysteroscopic, sampling and appropriate imaging. Progestin-based therapy is recommended, using a 52 mg levonorgestrel-releasing intrauterine device when feasible and/or continuous oral progestin; cyclic oral regimens are discouraged given lower regression rates. Close histologic surveillance every 3–6 months is required; non-response at 6–12 months, any progression, or failure to achieve complete response by ~24 months should prompt discussion of definitive surgery. For patients who are ineligible for surgery or decline it, progestin therapy may be continued with the same monitoring, and maintenance progestin can be considered when risk factors persist. Lifestyle counseling—weight loss and glycemic control—should accompany treatment [83,84,85].

Optimization of progestin therapy requires careful consideration of formulation, dose, delivery route, and lesion type. Across studies, histologic complete response rates for non-atypical EH/EIN range from ~67–94%, but recurrence remains substantial (~13.7–40.6%), underscoring the need for sustained therapy and lifestyle modification. Variability reflects differences in lesion type (EH vs. EIN), metabolic phenotype/obesity, adherence and device-related factors (e.g., expulsion), and sampling quality (preferably hysteroscopic). Route-specific endometrial exposure is critical: systemic and intrauterine delivery (LNG-IUS or continuous oral progestins) demonstrate superior efficacy, whereas vaginal micronized progesterone shows inconsistent results [22,79,82].

Responses to LNG-IUS and continuous oral progestins are heterogeneous across lesion type (EIN vs. non-atypical EH), metabolic phenotype, and adherence/device continuation. Recurrence can occur after initial histologic regression, particularly when risk factors persist or the device is discontinued. Accordingly, we emphasize close histologic surveillance and guideline-concordant stop rules and consider maintenance progestin in selected patients, with escalation to surgery for non-response or progression, per international guidance.

Current evidence supports a multifaceted approach to endometrial cancer prevention in obese women, combining targeted progestin therapy with metabolic interventions and lifestyle modifications. The LNG-IUS emerges as the preferred first-line option for most candidates, offering maximal efficacy with minimal systemic exposure, while combination strategies incorporating metformin and weight loss provide enhanced outcomes for complex cases.

## 4. Discussion and Conclusions

This review synthesizes current evidence on the mechanistic pathways linking obesity to endometrial carcinogenesis and motivates a shift toward preventive, metabolically focused clinical approaches.

Collectively, the evidence indicates that obesity orchestrates a convergent network of molecular perturbations that creates a pro-carcinogenic endometrial milieu. Specifically, obesity-induced hyperestrogenism, insulin resistance with IGF-1 axis activation, chronic low-grade inflammation, and dysregulated PI3K/AKT/mTOR signaling establish synergistic pathways that promote endometrial proliferation and malignant transformation.

For high-risk populations—including women with obesity, metabolic syndrome, or polycystic ovary syndrome—these insights support risk-stratified surveillance protocols that combine clinical phenotyping with emerging endocrine and molecular biomarkers. This personalized approach enables dynamic risk assessment and targeted risk-reduction measures and surveillance intensity based on individual metabolic profiles, rather than conventional one-size-fits-all screening paradigms.

The obesity–endometrial cancer nexus offers a compelling model for improving prevention strategies in gynecologic oncology. By reframing the endometrium as a metabolically responsive target organ, clinical practice can move from reactive, post-diagnostic interventions toward proactive, biology-informed risk reduction strategies. This preventive framework provides a credible and cost-effective path to reducing incidence and disease burden.

## 5. Limitations

The predominance of observational studies restricts causal inference, particularly regarding temporal relationships between molecular alterations and disease progression. Most mechanistic insights derive from cell culture and animal models that may incompletely recapitulate human adipose–endometrial tissue interactions. Study heterogeneity in population characteristics may limit generalizability of specific risk estimates. Long-term follow-up data for prevention interventions remain limited, particularly regarding sustained efficacy. Finally, underrepresentation of diverse ethnic populations constrains understanding of population-specific risk factors and intervention responses.

## 6. Future Research Directions

### 6.1. Lifestyle and Obesity Prevention

Future work should rigorously evaluate school- and community-based programs that build nutrition literacy, support regular physical activity, and promote adequate sleep—particularly among adolescent girls, whose hormonal status is shaped by diet from the years surrounding menarche. Studies should quantify long-term effects on adiposity, insulin resistance, menstrual regularity, and abnormal uterine bleeding, and identify scalable, cost-effective models for sustained risk reduction.

### 6.2. Translating Mechanisms into Prevention: Precision Risk Stratification and Monitoring

- Multi-omics risk models. Large prospective cohorts should examine interactions between obesity-related genetic polymorphisms (e.g., CYP19A1, PTEN, PIK3CA), adipokine expression profiles, and metabolomic signatures to delineate high-risk phenotypes requiring intensified preventive surveillance. Advanced machine learning approaches should integrate traditional risk factors with novel biomarkers, including circulating microRNAs, adipose tissue-derived exosomes, and inflammatory protein panels, to improve predictive accuracy.
- Longitudinal biomarker kinetics. Serial endometrial sampling studies should characterize temporal dynamics across the hyperplasia-to-carcinoma sequence—focusing on mTOR activation kinetics, STAT3 signaling, and adipokine fluctuations—as preventive readouts and to evaluate response to risk-reducing interventions (e.g., lifestyle modification), thereby informing the timing and intensity of surveillance.

### 6.3. Mechanism-Informed Target Discovery for Risk Reduction

Building on established molecular pathways, research priorities should include identifying prevention-relevant targets that normalize proliferative and inflammatory signaling in the obese endometrium. Focus on selective modulators of the PI3K/mTOR and STAT3 axes with endometrial specificity, and on adipokine-receptor modulators (leptin/adiponectin) that restore metabolic–hormonal homeostasis. Evaluate combinations that address convergent pathways, and platforms for local, uterus-confined exposure (e.g., drug-eluting intrauterine systems) to maximize efficacy while minimizing systemic effects. Chemopreventive candidates may include dual PI3K/mTOR modulators, STAT3 pathway modulators, and insulin-sensitizing agents, assessed explicitly for risk-reduction endpoints.

### 6.4. Mechanistic Model Development

Advanced tissue-engineering and organoid systems should characterize molecular crosstalk between adipose and endometrial tissues under obesogenic conditions. Three-dimensional co-culture models that incorporate human adipose tissue, endometrial organoids, and immune cells can recapitulate microenvironmental interactions during carcinogenic remodeling. Single-cell RNA sequencing and spatial transcriptomics should map cell-type-specific responses, interrogating extracellular-vesicle cargo, tissue-specific metabolic reprogramming, and epigenetic alterations, and linking these signatures to prevention endpoints and biomarker development.

## Data Availability

The original contributions presented in this study are included in the article. Further inquiries can be directed to the corresponding authors.

## Abbreviations

The following abbreviations are used in this manuscript:

AKT	Protein Kinase B
AdipoR1/2	Adiponectin Receptor 1 and 2
AMPK	AMP-Activated Protein Kinase
BAX	BCL2-Associated X Protein (pro-apoptotic)
BCL-2	B-Cell Lymphoma 2 (anti-apoptotic)
Bcl-xL	B-Cell Lymphoma–extra Large (anti-apoptotic Bcl-2 family)
CCND1	Cyclin D1 (Cyclin-Dependent Kinase 4 Regulatory Subunit 1)
CYP19A1	Cytochrome P450 Family 19 Subfamily A Member 1 (Aromatase)
DHEA	Dehydroepiandrosterone
EC	Endometrial Cancer
EH	Endometrial Hyperplasia
EIN	Endometrial Intraepithelial Neoplasia
ERα/ ERβ	Estrogen Receptor Alpha/ Beta
GLUT-4	Glucose Transporter Type 4
IGF-1	Insulin-Like Growth Factor 1
IL-6	Interleukin-6
IR	Insulin Receptor
IRS1/2	Insulin Receptor Substrate 1 and 2
JAK1/2	Janus Kinase 1 and 2
Ki-67	Marker of cellular proliferation (nuclear antigen expressed in cycling cells)
KRAS	Kirsten Rat Sarcoma Viral Oncogene Homolog
LNG-IUS	Levonorgestrel-Releasing Intrauterine System
MAPK	Mitogen-Activated Protein Kinase
MPA	Medroxyprogesterone Acetate
mTOR	Mechanistic Target of Rapamycin
NF-κB	Nuclear Factor κB
Ob-R	Leptin Receptor (Obesity Receptor)
PDPK1	3-Phosphoinositide-Dependent Protein Kinase-1
PIK3CA	Phosphatidylinositol-4,5-Bisphosphate 3-Kinase Catalytic Subunit Alpha
PIK3R1	Phosphatidylinositol-4,5-Bisphosphate 3-Kinase Regulatory Subunit 1
PI3K	Phosphatidylinositol-3-Kinase
PR	Progesterone Receptor
PTEN	Phosphatase and Tensin Homolog
pS6	Phosphorylated Ribosomal Protein S6
ROS	Reactive Oxygen Species
SAT	Subcutaneous Adipose Tissue
SHBG	Sex Hormone-Binding Globulin
S6K1	Ribosomal Protein S6 Kinase Beta-1 (p70S6K)
STAT3	Signal Transducer and Activator of Transcription 3
TNFR1	Tumor Necrosis Factor Receptor 1
TNF-α	Tumor Necrosis Factor-α
VAT	Visceral Adipose Tissue
VEGF	Vascular Endothelial Growth Factor
